# Real-Time Measure of the Lattice Temperature of a Semiconductor Heterostructure Laser via an On-Chip Integrated Graphene Thermometer

**DOI:** 10.1021/acsnano.3c01208

**Published:** 2023-03-08

**Authors:** Leonardo Viti, Elisa Riccardi, Harvey E. Beere, David A. Ritchie, Miriam S. Vitiello

**Affiliations:** † NEST, CNR - Istituto Nanoscienze and Scuola Normale Superiore, Piazza San Silvestro 12, 56127 Pisa, Italy; ‡ Cavendish Laboratory, University of Cambridge, Cambridge CB3 0HE, U.K.

**Keywords:** quantum cascade lasers, terahertz, graphene, thermometer, photoluminscence, cross-plane conductivity

## Abstract

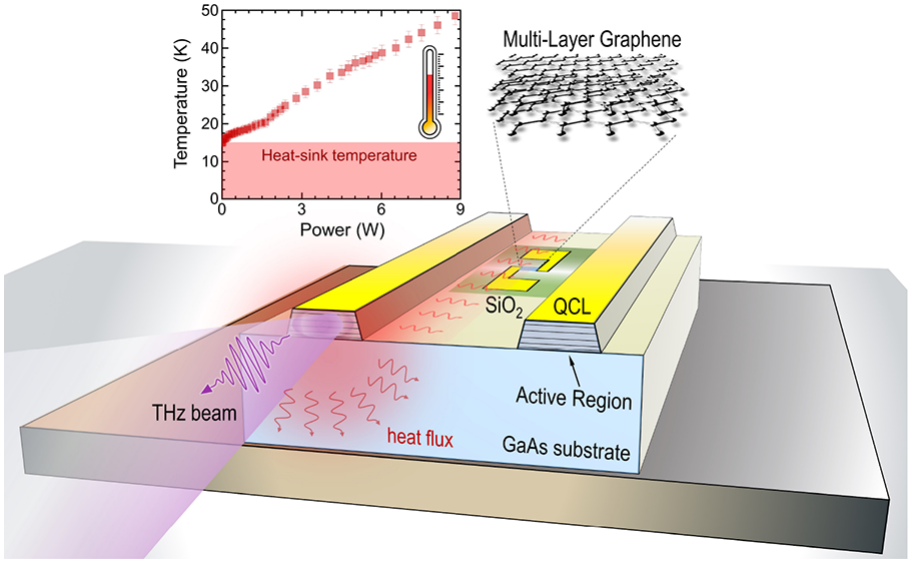

The on-chip integration of two-dimensional nanomaterials, having exceptional optical, electrical, and thermal properties, with terahertz (THz) quantum cascade lasers (QCLs) has recently led to wide spectral tuning, nonlinear high-harmonic generation, and pulse generation. Here, we transfer a large area (1 × 1 cm^2^) multilayer graphene (MLG), to lithographically define a microthermometer, on the bottom contact of a single-plasmon THz QCL to monitor, in real-time, its local lattice temperature during operation. We exploit the temperature dependence of the MLG electrical resistance to measure the local heating of the QCL chip. The results are further validated through microprobe photoluminescence experiments, performed on the front-facet of the electrically driven QCL. We extract a heterostructure cross-plane conductivity of *k*_⊥_= 10.2 W/m·K, in agreement with previous theoretical and experimental reports. Our integrated system endows THz QCLs with a fast (∼30 ms) temperature sensor, providing a tool to reach full electrical and thermal control on laser operation. This can be exploited, inter alia, to stabilize the emission of THz frequency combs, with potential impact on quantum technologies and high-precision spectroscopy.

Thermal management is a key requirement for the operation of any semiconductor laser^1^ because the major figures of merit, such as external quantum efficiency,^2^ lifetime, and power conversion efficiency^3,4^ strongly depend on the electron-acoustic phonon scattering rate,^5^ which determines the effective lattice temperature^6^ during device operation.^7^ An appropriate thermal management is also fundamental for applications deeply affected by temperature instabilities, e.g., those requiring a tight control of the phase and frequency jitter, such as quantum optics,^8^ metrology,^9^ and high-precision spectroscopy,^10^ or for near-field nanoimaging^11^ applications employing a semiconductor laser as pumping source or detector,^12,13^ since thermal instabilities may be reflected in fluctuations of the signal-to-noise ratio of the captured near-field signal amplitude and phases.^14,15^

This issue is even more severe in semiconductor heterostructure lasers. Here, heat extraction is hindered both by the large device thermal resistance,^16^*R*_T_*,* resulting from the low heat conductivity of the complex multilayer active regions (ARs),^17,18^ and by the poor thermal coupling between the AR and the heat-sink, as an effect of the mounting configurations and waveguide architecture.^19^

The heat conductivity is largely anisotropic, with both in-plane (*k*_//_) and cross-plane (*k*_⊥_) components being smaller than the thermal conductivities of the constituent bulk materials. This effect is more relevant in terahertz (THz) frequency quantum cascade lasers (QCLs),^20^ where the narrow separation between the intersubband levels involved in the lasing transition (5–20 meV) may require ultrathin and abrupt barriers, which enhance the rate of phonon scattering by interfaces, mainly in heterostructures with layer widths comparable or smaller than the phonon mean free path. These effects inherently inhibit the phonon transport and reduce the heat dissipation rate, as a consequence of the series of thermal boundary (Kapitza) resistances arising at each interface between materials of different thermal and mechanical properties.^16^ Diffusive scattering of phonons by interfaces^17^ in THz QCLs significantly reduces *k*_*//*_, while the reduction of *k*_⊥_ is mostly due to scattering by interface roughness and alloy disorder.

The assessment of the AR temperature during operation of QCLs, commonly dissipating a huge amount of electrical power, is hence necessary for modeling heat flow and local temperature distributions in QCLs.^20^ This is relevant for targeting room-temperature operation, a long-sought goal in the last two decades.

The most established method to monitor the AR heating during operation in mid-infrared (mid-IR)^21^ and THz^16,19,22,23^ QCLs is by means of microprobe band-to-band photoluminescence (PL) spectroscopy.^24^ This technique provides a way to indirectly determine the lattice temperature (*T*_L_) by measuring the redshift of the PL peak when the heat sink temperature (*T*_HS_) is increased,^25^ or when the AR is heated during laser operation (Joule heating). Importantly, microprobe PL spectroscopy gives a local measurement of *T*_L_ and of the electronic temperature;^23^ thus, it constitutes a powerful tool to infer the thermal resistance,^19,22^ the facet temperature profile,^16^ and the heat flow^25^ through the semiconductor heterostructure, giving access to the thermal dynamics within the device. However, the implementation of PL spectroscopy to determine QCL temperature during operation requires additional and complex experimental setups, making impractical a real-time monitoring of the local device heating during a specific application.

Alternative procedures to assess *T*_L_ in QCLs include the following: analysis of the dependence of the threshold current density on the heat sink temperature,^26^ or of the average laser power as a function of the duty cycle,^27^ transient interferometric thermal mapping^28^ or all-electrical techniques for monitoring the lattice temperature by measuring the heating of the laser metallic top contact.^29,30^ This latter method requires independent electrical access to four leads and at least four bonding wires to be collinearly placed on the QCL top contact. This entails complex bonding schemes^30^ and can be unpractical for specific device architectures, such as wire lasers^31^ or vertical emitting QCLs with two-dimensional photonic patterns,^32,33^ or for device applications requiring radio frequency coupling to the laser, e.g., injection-locked systems^34^ and dual-comb schemes.^35,36^

Here, we devise an integrated two-component system that allows monitoring the QCL lattice temperature during operation, by simply using one additional electrical access. The devised chip comprises a THz-frequency QCL and a graphene microthermometer, lithographically patterned adjacent to the laser ridge. Graphene field effect transistors proved to be highly efficient radiation sensors in the far-infrared.^37^ So far, graphene has been also incorporated/combined with THz QCLs to achieve gate-tunable spectral control,^38^ modulate the radiation intensity,^39^ or to stabilize the operation of frequency combs^40^ through its fast saturable absorption dynamics. Its rich physics,^41^ along with its versatility and fabrication flexibility, makes it a promising material platform for integration with different on-chip electronic^42,43^ and photonic^44,45^ solid-state architectures, such as CMOS,^46^ silicon on insulator (SOI),^47,48^ and SiN.^49^

Therefore, despite the relatively weak temperature coefficient of resistance (TCR = d*R*/*R*d*T* ≈ −1%/K), graphene is a promising candidate for the realization of integrated thermometers. Negative TCR thermistors are commonly used as on-chip temperature sensors for the thermal stabilization of near-infrared laser diodes in combination with Peltier coolers.^50^ Thus, depending on the required operating temperature range, two-dimensional (2D) materials, preferably with high TCR (e.g., WS_2_^51^ or Mo_*x*_W_1–x_S_2_^52^), could be employed as on-chip thermistors in combination with electrically pumped semiconductor sources, such as mid-infrared GeSn/SiGeSn heterostructure lasers.^53^

## Results and Discussion

THz-frequency QCLs, based on a bound-to-continuum GaAs/Al_0.15_Ga_0.85_As active region,^4^ emitting at a wavelength λ = 110 μm (2.7 THz), are fabricated on single plasmon waveguides^54^ following the procedure described in Methods. A 500 nm thick SiO_2_ layer, covering a surface of 300 × 800 μm^2^, is deposited by Ar sputtering on the bottom doped layer between each pair of laser bars to host and electrically isolate the integrated thermometers.

**Figure 1:**
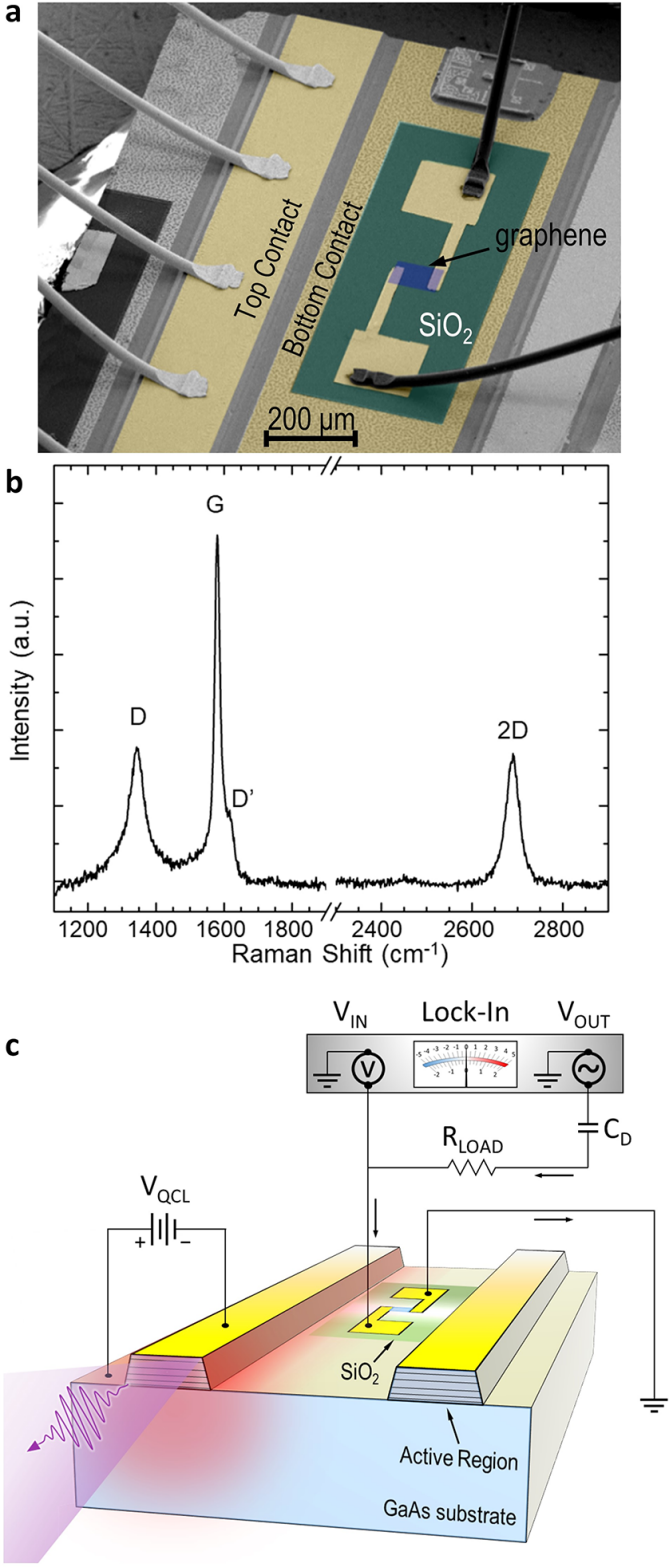
Device architecture. (a) Scanning electron microscopy image of the fabricated chip. (b) Raman spectrum of transferred MLG on the QCL chip. (c) Schematic diagram of the devised system: the graphene thermometer is integrated on-chip between the ridges of the fabricated QCLs. The thermistor resistance (R_gr_) is real-time monitored by means of a lock-in technique. Arrows indicate the current flow through the voltage divider.

The integrated thermometer comprises a stack (7 layers) of single-layer graphene (SLG). The resulting multilayer graphene (MLG) film is easier to manipulate and more stable during the transfer process with respect to SLG. The performance of different thermometers as a function of the number of layers is analyzed and reported in the Supporting Information (Figure S1). The TCR increases as a function of thickness, especially for thicknesses larger than 5 layers. Using a 5/7-layer graphene results in the best compromise between bolometer performance and sample robustness that increase with thickness, and fabrication complexity and sample quality that degrades after a large number of wet-transfer processes. Commercially available (Graphenea Inc.) SLG samples, grown on Cu via chemical vapor deposition (CVD), are sequentially transferred on top of each other using a wet-transfer technique: A4-950K poly(methyl-methacrylate) polymer (PMMA) is spin coated at 2000 rpm on the surface of an SLG sample and then placed in a solution of 1 g of ammonium persulfate (1 g diluted in 40 mL of DI water) to etch the Cu substrate. Once the Cu etching is complete, the PMMA-SLG film is transferred in a beaker with DI water and then lifted with a second Cu-graphene square to obtain an MLG sample. The sample is left to dry, and finally the PMMA is removed with acetone. The final Cu substrate is eventually etched, and MLG is transferred on top of the QCL device. MLG channels of 30 × 100 μm^2^ are lithographically defined on the SiO_2_ patches, etched with oxygen plasma, and connected to two Cr/Au (10/150 nm) electrodes. Finally, the GaAs substrate is lapped down to a thickness of ∼200 μm and back coated with a 10/50 nm Cr/Au metallic layer to improve thermal coupling with the copper bar. Laser bars 2.1 mm long and 200 μm wide are cleaved, mounted on a copper bar through a dedicated thermal InAg alloy (97%: 3%), and wire bonded. A scanning electron microscopy (SEM) image of a prototypical device is shown in Figure 1a. The position of the graphene thermometer has been preliminarily defined after performing thermal simulations of the whole QCL structure (see Figure S2), which show a marginal thermal gradient (<1 °C) along the laser ridge.

Micro Raman experiments are performed on the transferred MLG using a confocal Raman spectrometer (Horiba, Explora Plus) equipped with a 532 nm laser in backscattering configuration. We use a 100× objective producing a laser spot size of ∼0.5 μm. The Raman spectrum of the MLG thermometer, plotted in Figure 1b, shows, as expected, two main Raman peaks: the G (position POS(G) = 1580 cm^–1^, full width at half-maximum fwhm = 20 cm^–1^) and 2D (POS(2D) = 2690 cm^–1^, fwhm = 37 cm^–1^) peaks.^55^ The 2D band is always single-peaked, less intense with respect to the G peak (∼), and broad, indicative of rotationally disordered MLG.^56^ In addition to these bands, the D (POS(D) = 1344 cm^–1^, fwhm = 67 cm^–1^) and D′ (POS(D′) = 1616 cm^–1^, fwhm = 24 cm^–1^) Raman peaks are observed. These features, coming from defect-assisted Raman processes,^57,58^ reflect the disorder introduced during the multiple graphene-transfer steps.

The integrated laser-thermometer system (Figure 1c) is engineered to drive the QCLs and the graphene thermometer independently. The thermometer exploits the temperature-dependent resistance of the graphene film *R*_gr_, which is monitored with a lock-in amplifier (time constant 30 ms) over a voltage dividing circuit: the lock-in signal is *V*_IN_ = *V*_OUT_*R*_gr_/(*R*_Load_ + *R*_gr_), where *V*_OUT_ is a sine wave with amplitude 40 mV and frequency *f*_mod_ = 343 Hz, generated by the lock-in itself, and *R*_Load_ = 3.3 kΩ is chosen to be close to *R*_gr_. A capacitor *C*_D_ = 1 μF is added to the circuit to act as a *dc*-block on the thermometer line. Therefore, this architecture is able to keep continuous track of temperature variations through the dependence *R*_gr_(*T*), with a time scale given by the lock-in time constant.

**Figure 2:**
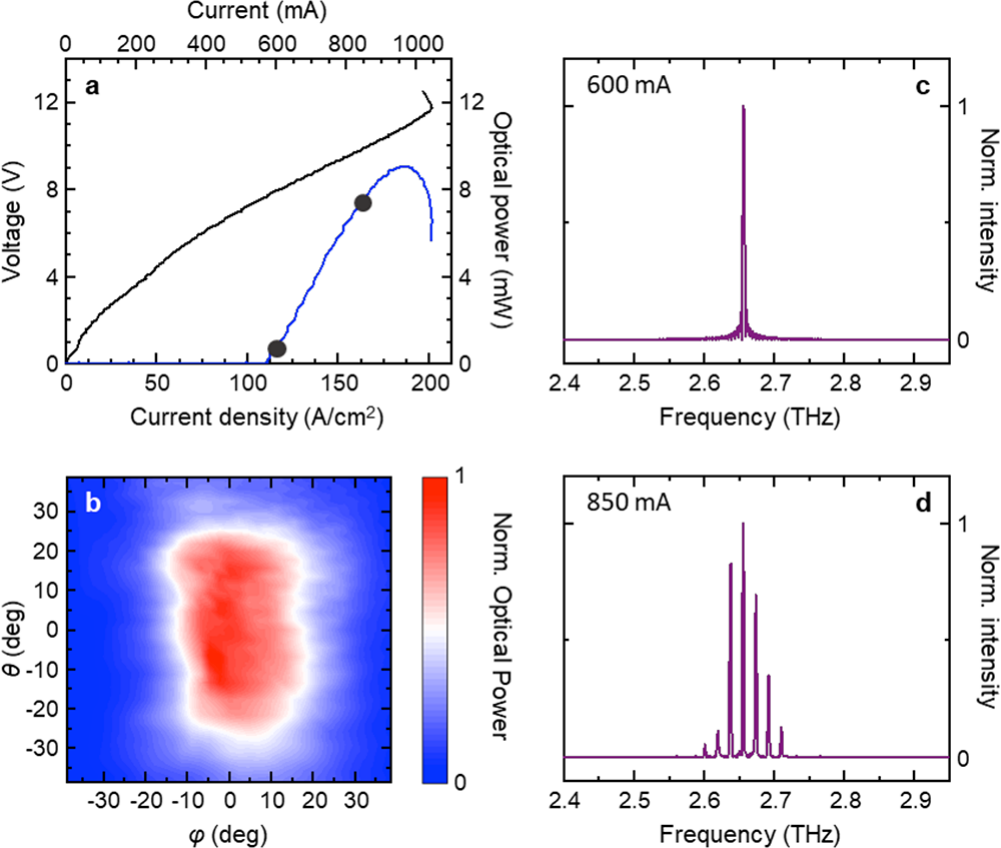
QCL characterization. (a) Light–current–voltage (LIV) characteristics of the investigated QCL, measured at *T*_HS_ = 15 K in pulsed mode with pulse width 1 μs and pulse repetition rate 100 kHz. (b) Far-field beam pattern measured with a pyroelectric sensor having a sensitive area of 7 mm^2^. (c and d) FTIR spectra (resolution 0.075 cm^–1^) collected in vacuum at *T*_HS_ = 15 K with driving currents 600 mA and 850 mA, respectively, corresponding to the black dots on the *I*–*V* characteristic of panel a.

We first characterize the QCL optical and electrical performance. Samples are mounted in a helium flow cryostat, and *T*_HS_ is monitored by a silicon diode sensor and varied in the range 6–300 K, through a tunable heater. Figure 2a shows the voltage–current density (*V*–*J*) and the light–current density (*L*–*J*) characteristics acquired while driving the QCL in pulsed mode (10% duty cycle, repetition rate 100 kHz) at *T*_HS_ = 15 K. The optical signal is further modulated with a 33 Hz square envelope to allow for lock-in detection. The QCL has a threshold current density *J*_th_ = 115 A cm^–2^ and delivers a maximum output power of 9 mW when biased at *V*_QCL_ = 10.3 V, *I*_QCL_= 900 mA. This optical power is calibrated with a power meter (TK Instruments, aperture 55 × 40 mm^2^) and corrected to account for the transmittance (25% at ∼3 THz) of the high-density polyethylene cryostat window. The far-field intensity distribution is collected with a pyroelectric detector (Figure 2b), raster scanned on a spherical surface of radius ∼5 cm, centered on the QCL front facet. The measured beam divergence (Figure 2b) is Δθ × Δφ = 25° × 10°.

Figures 2c,d show the Fourier transform infrared (FTIR) emission spectra, measured in a vacuum spectrometer (Bruker Vertex V80, resolution 0.075 cm^–1^) at *T*_HS_ = 15 K when the QCL is driven in CW at 600 mA and 850 mA, respectively: the laser is single-mode (2.56 THz) above threshold and becomes progressively multimode when the current is increased, spanning a 110 GHz frequency range (2.60–2.71 THz).

**Figure 3:**
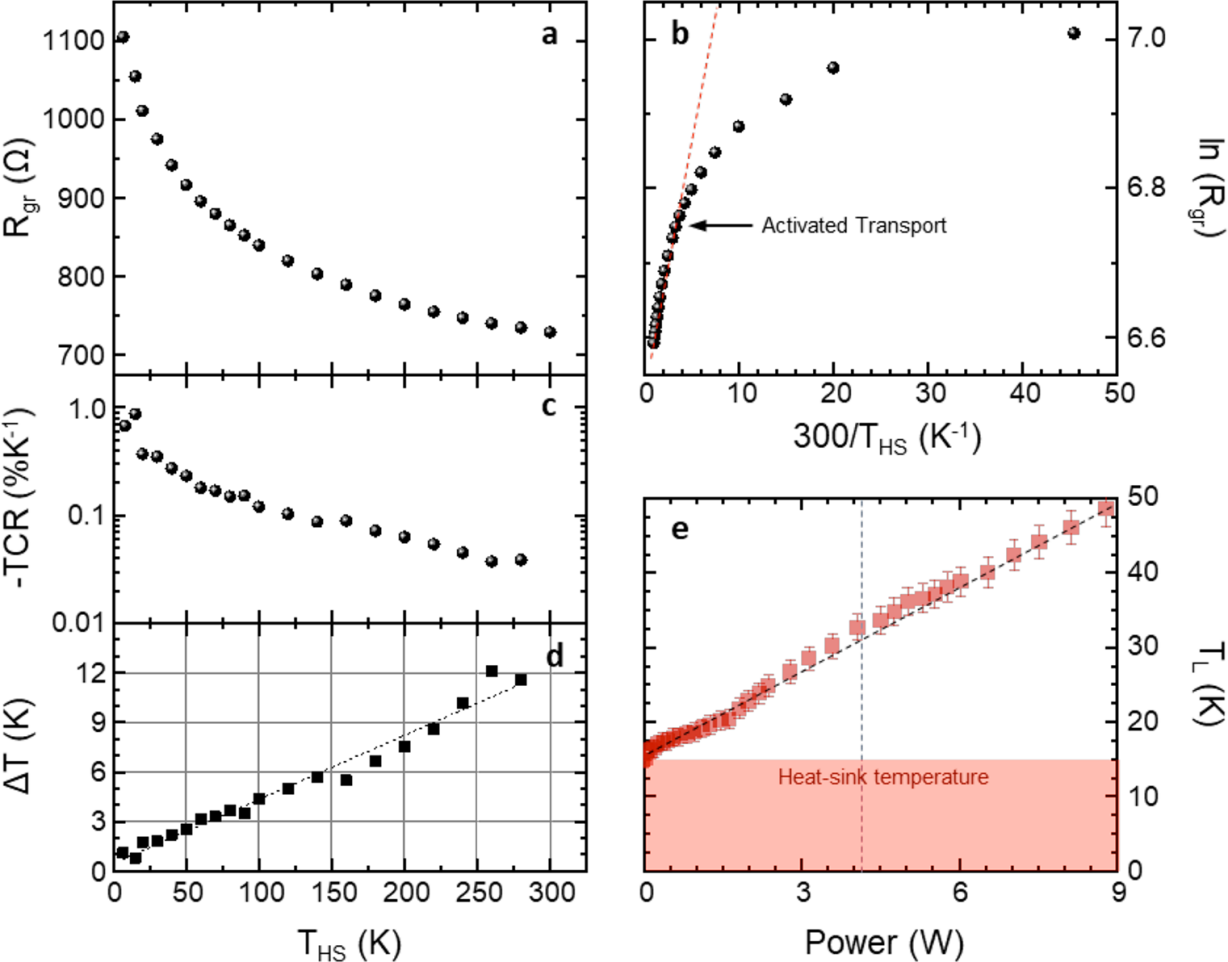
(a) Calibration curve *R*_gr_(*T*_HS_) of the graphene thermometer, recorded by changing *T*_HS_ between 6.6 and 300 K. (b) Arrhenius plot of *R*_gr_(*T*_HS_), showing thermally activated transport for *T*_HS_ > 80 K. (c) Temperature coefficient of resistance, extracted from *R*_gr_(*T*_HS_). (d) Thermometer accuracy as a function of temperature. The dashed line is a linear fit to the data: Δ*T* = *a* + *b* × *T*_HS_, with *a* = 0.48 K and *b* = 0.039. (e) Lattice temperature measured by the graphene thermometer during laser operation. The error bars are calculated from the accuracy plot in d. The black dashed line is a linear fit to the data. The vertical dashed line indicates the laser threshold power.

We then calibrate the graphene thermometer by measuring the thermoresistance characteristics, i.e., the dependence of *R*_gr_ from *T*_HS_, as shown in Figure 3a. *R*_gr_ decreases when the heat-sink temperature is increased, indicating a negative TCR and a thermally activated electrical transport, in agreement with previous findings on graphene thermistors.^59,60^ In the high-temperature limit (*T*_HS_ > 80 K), *R*_gr_ follows an exponential dependence on *T*_HS_, given by the Arrhenius equation.^60^*R*_gr_ = *R*_0_ exp(*B*/*T*_HS_), where *R*_0_ is the resistance at infinite *T*_HS_ and *B* = *E*_a_/2*k*_B_ is the thermal index, with *E*_a_ activation energy in the graphene layer and *k*_B_ Boltzmann constant. Figure 3b shows the Arrhenius plot of ln(*R*_gr_), as a function of 300/*T*_HS_. From the linear fit to the data, in the range *T*_HS_ > 80 K, we find *R*_0_ = 698 ± 1 Ω, *B* = 17.6 ± 0.9 K, and *E*_a_ = 3.0 ± 0.1 meV; these thermal figures of merit are close to those measured in printed multilayer graphene films, at room temperature.^59^ The *R*_gr_(*T*_HS_) curve can be used to determine the sensitivity of the graphene thermistor in terms of the TCR figure of merit: |TCR| ∼ 1% K^–1^ for *T*_HS_ < 30 K and progressively decreases to <0.1% K^–1^ for *T*_HS_ > 100 K (Figure 3c). We then use TCR(*T*_HS_) to estimate the accuracy (deviation from true temperature) of the graphene thermometer by calculating the standard deviation Δ*T* stemming from the instrumental error Δ*R*_gr_ in the *R*_gr_(*T*_HS_) calibration curve. Results are shown in Figure 3d: the accuracy worsens (increases) linearly as a function of temperature, being < ±2.5 K for *T* < 50 K and reaching ±12 K at room temperature. The accuracy increase is caused by the reduction of the thermistor TCR at high temperature.

We then use the calibration curve *R*_gr_(*T*_HS_) to monitor the temperature of the graphene thermometer (*T*_G_) during laser operation. The QCL is driven in continuous wave (CW), while *T*_HS_ is kept at 15 K. Figure 3d shows *T*_G_ as a function of the electrical power (*P*_e_) dissipated in the QCL. *T*_G_ grows linearly from *T*_HS_ = 15 K to a maximum of 48 K, suggesting that the graphene thermometer is effectively measuring the device temperature, whose dependence from the electrical power is expected to be linear.^23^ From the linear fit to the *T*_G_(*P*_e_) plot, we obtain a slope *R*_TG_ = d*T*_G_/d*P*_e_ = 3.86 K W^–1^.

We then compare *T*_G_ extracted with the graphene thermometer with the lattice temperature measured by means of a microprobe PL technique (*T*_PL_). For this experiment, the QCL is mounted in the cold unit of a He flow microcryostat (Janis, ST-500-UC), with the laser facet facing the cryostat quartz window (1.5 mm thick). The cryostat is fixed below the objective (50×, long-working-distance = 15 mm) of a confocal Raman spectrometer (Horiba, Explora Plus), and the ∼1 μm focal spot is aligned with the center of the QCL facet by means of a motorized stage.

PL spectra are acquired using a 1800 grooves/mm grating and a 638 nm laser whose optical power density is attenuated to <10 kWcm^–2^, thus keeping the laser-induced electron heating below ∼5 K.^22^ The laser excitation provides the valence band holes needed to probe the electronic population via interband radiative recombination. We use the facet temperature as a close estimate of the internal one due to the absence of nonradiative surface electron–hole recombination processes in unipolar devices.

**Figure 4:**
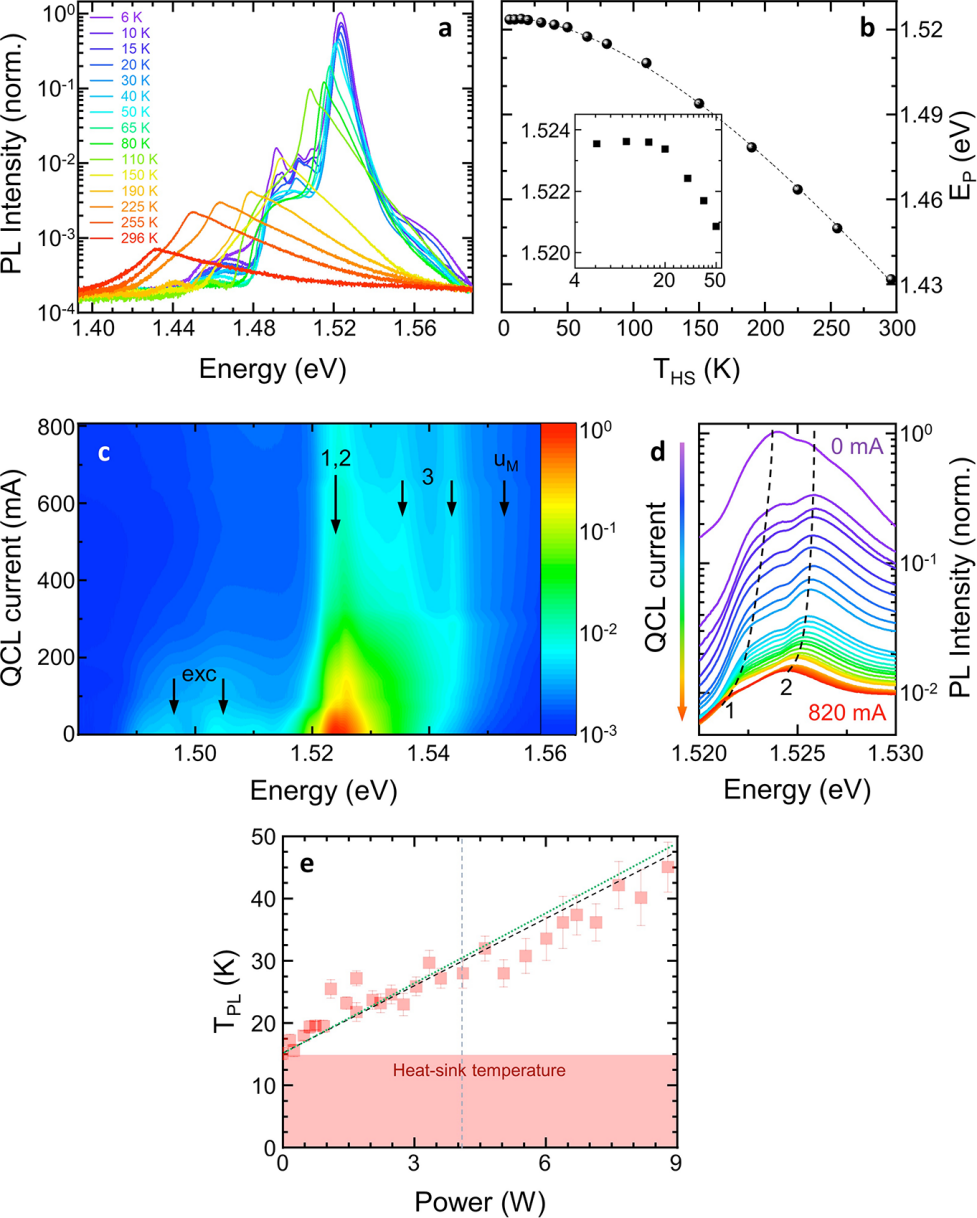
(a) PL spectra acquired by probing the QCL facet at zero current, while varying *T*_HS_ from 6.0 to 296 K, with confocal hole size *h* = 100 μm. To properly convert the data recorded in units of wavelength to units of energy, spectra were rescaled by applying the Jacobian transformation.^66^ (b) *E*_P_(*T*_HS_) calibration curve (dots). The dashed line is a fit to the experimental data by using the Varshni’s equation. Inset: experimental data in the *T*_HS_ range between 4 and 50 K. (c) Color map of the PL spectra from the laser facet, measured while driving the QCL in CW at increasing currents from 0 mA to 820 mA. The arrow labeled “1,2” represents the injection doublet position, which shifts when the QCL heats up. (d) Main PL peaks acquired at different QCL currents. Dashed lines are a guide for the eye. (e) Lattice temperature measured with the PL technique during laser operation. The black dashed line is a linear fit to the data, corresponding to a thermal resistance of 3.62 KW^–1^. The green dotted line represents the fit to the data obtained with the graphene thermometer (from Figure 3d), reproduced here for direct comparison. The vertical dashed line indicates the lasing threshold.

Figure 4a shows PL spectra recorded at the center of the QCL facet while keeping the QCL unbiased and sweeping *T*_HS_ in the range 6.0–296 K. Each spectrum shows a main peak, located at an energy *E*_p_, ascribed to band-to-band transitions between levels in the injector miniband, where the vast majority of electrons sit, and valence subbands, as typically retrieved in bound-to-continuum active region designs. For *T*_HS_ < 20 K, the PL spectra display four peaks, corresponding to free excitons and impurity-bound excitons.^61,62^ As the temperature is increased, the main peak redshifts as an effect of the temperature-induced change of the GaAs energy gap; this redshift can be used as a thermometric property to extract the calibration curve (Figure 4b), i.e., the *E*_p_ value measured while varying *T*_HS_. We fit the experimental calibration curve with the semiempirical Varshni equation^63^*E*_P_(*T*_HS_) = *E*_P_(0) – α*T*_HS_^2^/(β + *T*_HS_), which is typically used to describe the band gap shrinkage with temperature, obtaining *E*_P_(0) = (1.524 ± 0.0003) eV, α = (7.5 ± 0.5) × 10^–4^ eV K^–1^ and β = (405 ± 45) K. The value of *E*_P_ can in turn be used to extract the local lattice temperature (*T*_PL_) during laser operation, provided that the calculated field-induced shift of the confinement energies in the QCL active region is properly considered.^21^ The field-effect correction is relevant only below the threshold for current-injection.

We then collect a set of PL spectra while varying the QCL driving current, hence *P*_e_. *T*_HS_ is kept fixed at 15 K. When the laser is driven in CW, the active region is Joule-heated and *E*_P_ redshifts as *P*_e_ increases. The color-map of the PL intensity as a function of energy and QCL driving current is reported in Figure 4c. Here, the injector doublet^23^ is marked with labels “1” and “2”; the excited laser level excitons^4^ are marked with label “3”; the upper miniband,^64^ which becomes progressively populated with the applied electric field, is marked with “u_M_”; and the low-energy peaks at low QCL currents correspond to free excitons and impurity-bound excitons (marked with “exc”). The position of the main PL peak shifts with increasing current, as described in Figure 4d, which shows the PL spectra in the energy range between 1.52 and 1.53 eV, collected while changing *P*_e_ from 0 to 8.8 W. The position of the lowest-energy peak is calculated by fitting the whole spectrum with a combination of Lorentzian and Lorentzian-exponential functions.^65^ This procedure is repeated for each value of the QCL electrical power to determine the dependence of *E*_P_, and in turn *T*_PL_, from *P*_e_ (Figure 4e**)**. The lattice temperature, extracted from micro-PL at the center of the laser facet, increases linearly with *P*_e_, with *R*_TL_= 3.62 KW^–1^, similar to the value measured by the graphene thermometer (*R*_TG_). The direct comparison between the error bars in Figures 3e and 4e, measured with the graphene thermistor and with the microprobe PL technique, respectively, demonstrates that the on-chip thermometer provides a better accuracy with respect to the PL method for temperatures <50 K.

**Figure 5:**
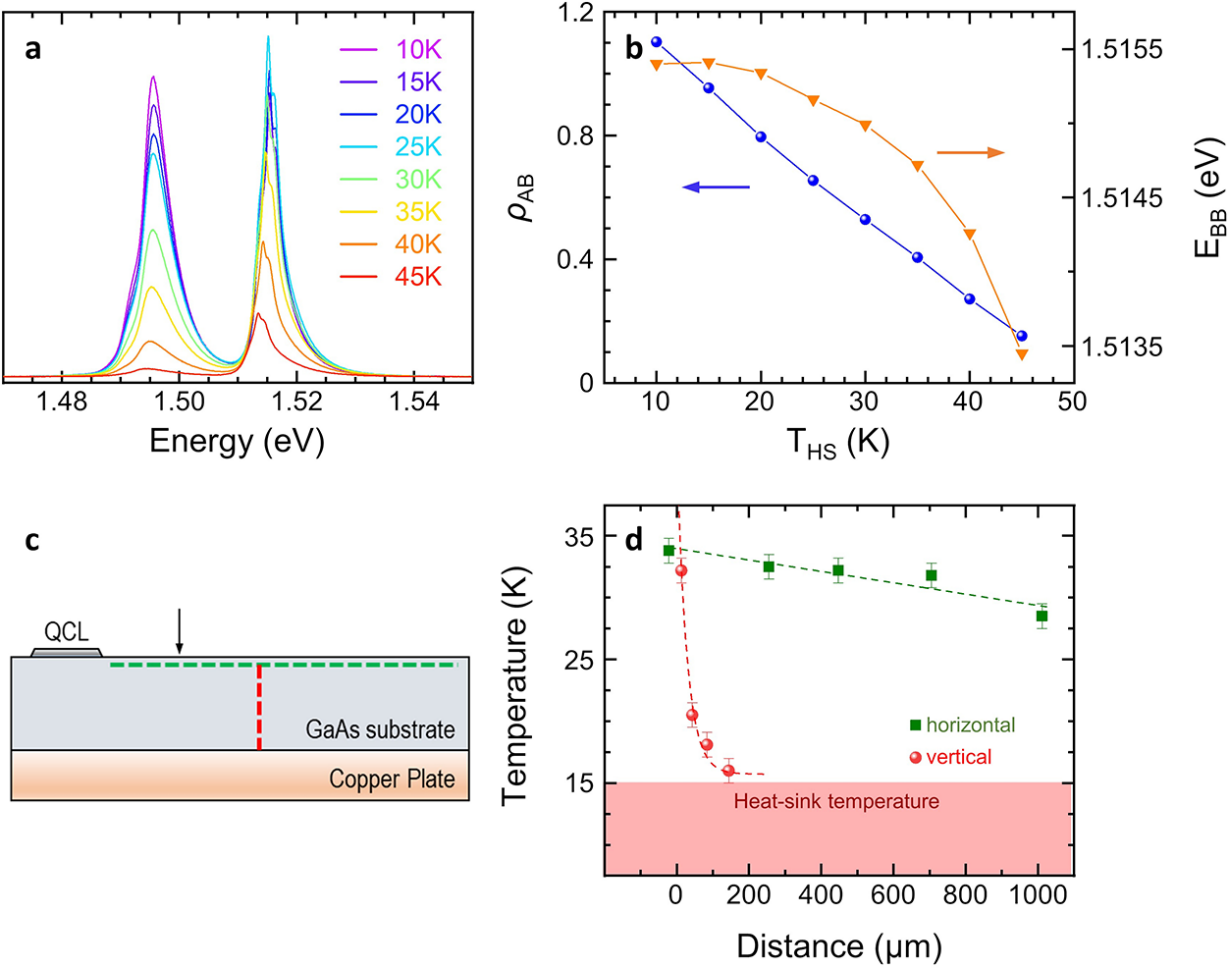
(a) Microprobe PL spectra, acquired on the Si-doped GaAs substrate, while changing *T*_HS_ from 10 to 45 K (laser wavelength 638 nm, confocal hole size *h* = 100 μm). (b) ρ_AB_ and *E*_BB_ plotted as a function of *T*_HS_. (c) Schematic view of the surface plane investigated by PL spectroscopy. The arrow indicates the projection of the graphene thermometer on the GaAs substrate front-surface. The green and red dashed lines indicate the profiles along which the PL spectra are recorded. (d) *T*_PL_ measured at different positions on the substrate. Green squares (red circles) indicate the results obtained while moving along the green (red) line in panel c. Distances are measured from the lower-right corner of the QCL front-facet along the horizontal direction and from the GaAs substrate top-surface along the vertical direction. The dashed lines are fits to the data.

We then monitor, via micro-PL spectroscopy, the substrate temperature during laser operation. Figure 5a shows characteristic microprobe PL spectra of the Si-doped GaAs substrate,^67^ measured while sweeping *T*_HS_ in the range of 10–45 K. The peak at ∼1.515 eV corresponds to the band-to-band (B–B) luminescence of GaAs, i.e., the energy gap in the bulk, whereas the peak at ∼1.495 eV is attributed to a band-to-acceptor (B–A) luminescence due to residual carbon impurities, which typically create an acceptor level in GaAs.^67^ The intensity ratio (ρ_AB_) between the B–A and B–B PL peaks is strongly dependent on the local temperature, providing an optimal observable to determine it. Figure 5b compares the *T*_HS_ dependencies of the B–B energy (*E*_BB_) and ρ_AB_. When *T*_HS_ is increased from 10 to 45 K, ρ_AB_ decreases almost linearly from 1.1 to 0.15, with a slope −0.027 K^–1^, whereas *E*_BB_ decreases by just 2 meV, with an initial flat region that hampers a correct evaluation of *T*. We therefore exploit the calibration of ρ_AB_(*T*_HS_) to estimate the local temperature *T*_PL_ at different positions in the GaAs substrate when *P*_e_ = 9 W (dashed lines in Figure 5c). When moving from the wafer top-surface along the vertical direction (red line), *T*_PL_ decreases exponentially, with a decay length ∼35 μm (Figure 5d), approaching *T*_HS_ close to the interface between the substrate and the copper plate, where *T*_PL_ = 16.2 K. On the other hand, while moving along the direction parallel to substrate surface (green line, 15 μm below the surface), *T*_PL_ slowly decreases following a linear trend with a slope of −4.6 K mm^–1^. From the fits to the data, we can infer a temperature ∼40 K on the substrate top-surface at a position ∼200 μm from the ridge corner (black arrow in Figure 5c), corresponding to the projection of the graphene thermometer on the chip front-surface. The 9 K discrepancy with the value reported in Figure 3d (49 K) can be ascribed to the fact that the graphene thermometer is close to the center of the chip, whereas the PL measurement is probing a boundary of the GaAs substrate, which is expected to be colder (see Supporting Information, Figure S2).

The measured substrate heating reveals that the temperature drop in the GaAs wafer plays a relevant role in the thermal management of the device. Indeed, by using the results in Figure 5d, we can estimate the temperature at the interface between the active region and the lower cladding *T*_b_ = 42.5 K, while at the facet center *T*_PL_ = 48.5 K, with a bottom-to-center temperature difference Δ*T*_bc_ = 6 K. Thus, ∼70% of the temperature increase with respect to *T*_HS_ takes place in the substrate. Assuming that the lateral heat extraction is suppressed in the laser ridge^29^ and using a one-dimensional model along the growth axis, we derive the relation Δ*T*_bc_ = 1/4·(*P*_e_*R*_L_), from which we estimate the absolute thermal resistance of the QCL lattice *R*_L_= 2.7 K/W and a cross plane thermal conductivity *k*_⊥_= *d*/(*R*_L_·*A*) = 10.2 W/m·K, which is in agreement with previous results on similar heterostructures.^30,68^ We can thus decompose the total thermal resistance *R*_TL_ into the contributions of the substrate and the active region, *R*_TL_ = *R*_S_ + *R*_L_/4, from which we estimate an absolute thermal resistance of the GaAs wafer *R*_S_ = 2.95 K/W.

## Conclusions

Our experiments demonstrate that the on-chip integrated graphene thermometer provides a reliable evaluation of the active region lattice temperature during laser operation, allowed by the optimal thermal coupling between the laser ridge and the electrode between the ridges on which the measurement relies.

The value of *T*_G_, measured by the graphene thermometer, is retrieved on a time scale given by the lock-in integration time constant (30 ms), enabling the active control of the QCL temperature by measuring *R*_gr_(*T*), rather than by the conventional control of *T*_HS_. This can be exploited to directly stabilize *T*_L_ and can be applied in different scenarios, e.g. in the realization of frequency stable THz QCL frequency combs, where the dependence of the intermode beatnote frequency Δν on the active region temperature (∼ −5 MHz K^–1^)^69^ can lead to drifts in Δν, which are unavoidable if the temperature feedback to the control system is given by *T*_HS_.

In conclusion, we have demonstrated integrated graphene thermometers, based on transferred large-area (∼1 × 1 cm^2^) MLG (commercially available), and monolithically mounted on a QCL chip. The scalability of the fabrication technique can allow, as a future perspective, the realization of multiple thermometers, ideally connected to multiple local heaters and multiple QCL ridges for the parallel stabilization of the temperature all over the chip. We have here adopted graphene for the ease of transfer and manipulation; however, the use of other 2D materials,^70^ above all with higher TCR^51,52^ is also promising and could provide a larger temperature sensitivity thanks to steeper thermoresistance characteristics. Owing to the wide spectrum of thermoresistive 2D materials,^51,52^ to the advancement of synthesis and transfer techniques,^71^ and to the general character of the proposed approach,^50^ it can find application in several electrically pumped radiation sources operating at different temperatures, such as THz and mid-IR QCLs, difference frequency generation QCL,^72^ and infrared light-emitting diodes in single and large array configurations.

## Methods

### Fabrication Procedure

The *d* = 12 μm thick active region is embedded between a doped ([Si] = 2.0 × 10^18^ cm^–3^) 700 nm thick GaAs bottom layer and a doped ([Si] = 5.0 × 10^18^ cm^–3^) 80 nm thick GaAs top contact layer. The laser bars are defined by means of optical UV lithography and wet etching of the active region (H_2_SO_4_:H_2_O_2_:H_2_O with proportion 5.5:4.5:25 mL). Top and bottom AuGe/Au (80/100 nm on the top and 80/150 nm on the bottom) ohmic contacts are then lithographically defined, thermally evaporated, and annealed at 400 °C.

## Supporting Information Available

Properties of the MLG thermometers as a function of the number of layers (section S1) and thermal simulations of the quantum cascade laser chip and cold-unit (section S2) (PDF)
