# Supplementary material for: Real-Time Measure of the Lattice Temperature of a Semiconductor Heterostructure Laser via an On-Chip Integrated Graphene Thermometer
Source: ACS Nano. 2023 Mar 8;17(6):6103–12. doi: 10.1021/acsnano.3c01208 (PMC10062027; doi:10.1021/acsnano.3c01208)
Supplement: Supplementary file 1 — nn3c01208_si_001.pdf [file nn3c01208_si_001.pdf]

Supporting information

Real-time measure of the lattice temperature of a semiconductor heterostructure laser via an on-chip integrated graphene thermometer

Leonardo Viti,[1+] Elisa Riccardi,[1+] Harvey E. Beere,[2] David A. Ritchie,[2] Miriam S. Vitiello[1*]

[1] *NEST, CNR - Istituto Nanoscienze and Scuola Normale Superiore, Piazza San Silvestro 12, 56127, Pisa, Italy*

[2] *Cavendish Laboratory, University of Cambridge, Cambridge CB3 0HE, UK*

[+] Authors contributed equally to the work

S1. Properties of the MLG thermometers as a function of the number of layers

We investigate the performance of different MLG-based thermometers as a function of the thickness of the employed MLG. Several devices with thicknesses ranging between 1 and 7 graphene layers were realized. The MLG geometry is the same as those integrated on the sides of the QCL (30×100 µm) in the main text (**Figure S1a**). To fabricate the thermistors, we use a silicon substrate covered with 300 nm of SiO_2. The substrate backside is evaporated with gold to improve the thermal contact with the copper heat sink. Thermometers have a room temperature resistance ranging from 600 Ω to 1500 Ω. The calibration of the devices as a function of T_{HS}, stable over multiple temperature cycles, is shown in **Figure S1b**. All the devices show a negative TCR.

For an easier comparison, **Figure S1d** shows the resistance normalized to the resistance at infinite temperature R_0 extracted from the Arrhenius fit (**Figure S1c**), from which we also derive the activation energy E_a, whose values are reported in the table. Finally, the TCR is calculated and plotted as a function of T_{HS} (**Figure S1e**), showing a clear increase with the number of layers, from 0.4 $\%K^{-1}$ (SLG) to 1.9 $\%K^{-1}$ (7LG) at 15 K (**Figure S1f**). This trend indicates that a thicker MLG sample is advantageous not only for the robustness of the graphene film, but also for the thermal properties of the thermometers.

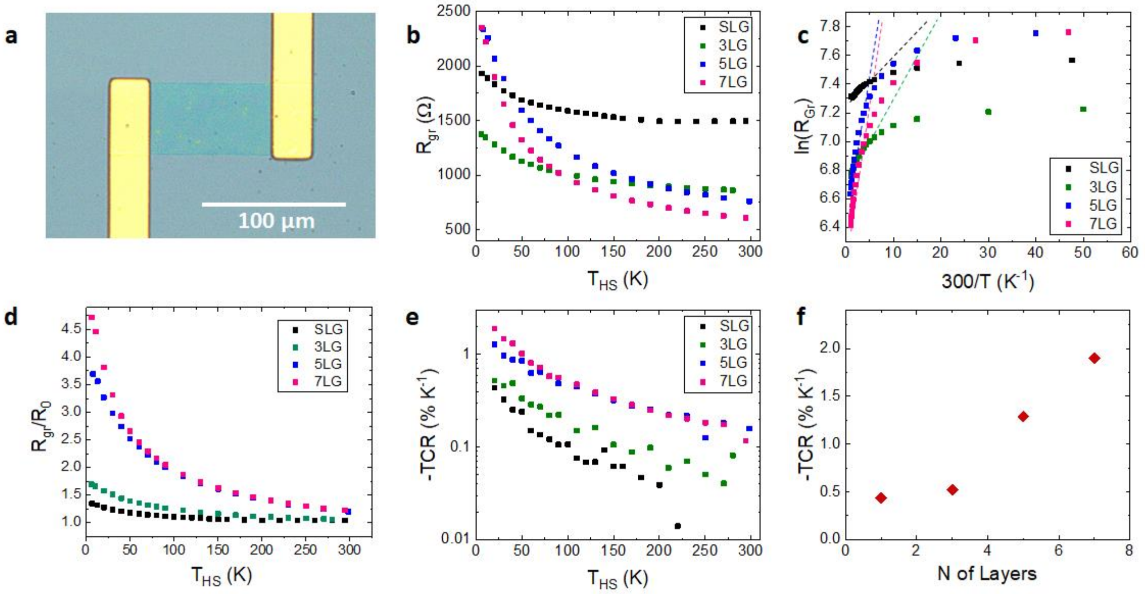

Arrhenius coefficients	1 Layer	3 Layers	5 Layers	7 Layers
R_0 (Ω)	1436	810	633	498
E_a (meV)	1.6	3.5	11.5	11.7

Figure S1. (a) Optical microscope image of a fabricated MLG bolometer. (b) Calibration curves $R_{gr}(T_{HS})$ of the graphene thermometers made with 1-3-5-7 layers of graphene, recorded by changing T_{HS} between 6.6 K and 298 K. (c) Arrhenius plot of $R_{gr}(T_{HS})$ for the different devices. The result of the fitting procedure is reported in the table. (d) $R_{gr}(T_{HS})$ of the graphene thermometers normalized to the high-temperature resistance R_0 extracted from the Arrhenius fits. (e) Temperature coefficient of resistance extracted from $R_{gr}(T_{HS})$. (f) TCR at 15 K as a function of the number of layers.

S2. Thermal simulations of the quantum cascade laser chip and cold-unit.

In this section, we report on the thermal simulations of the cryostat cold-unit and QCL chip, performed with a commercial software (COMSOL Multiphysics). The simulation geometry includes the oxygen-free copper mount where the QCL is mounted during operation (**Figure S2a**). The heat-sink temperature is kept constant at 15 K and the system is excited by an electrical power applied to the QCL ridge. **Figure S2b** presents a SEM image of the device, where the red dashed line indicates the line on the sample surface along which the temperature profile in **Figure S2c** is evaluated, showing a small temperature variation in the direction parallel to the QCL, with a maximum at the center of the ridge length. This result indicates the optimum position to place the thermometer to obtain maximum accuracy.

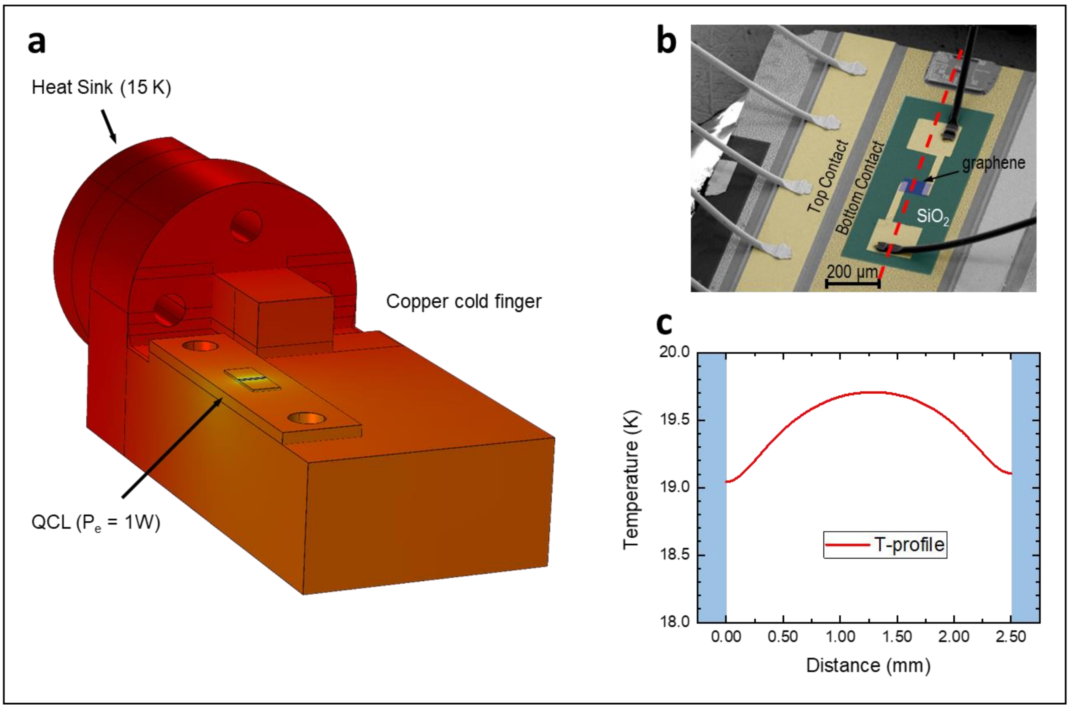

Figure S2. (a) Thermal simulation of the cryostat cold-unit, when the QCL is biased with an electrical power of 1 W and with T_{HS} = 15 K. (c) Temperature profile as a function of distance, taken on the substrate surface, in the direction parallel to the QCL ridge, along the dashed red line in (b). The blue shaded areas are regions outside the QCL ridge.
